# Simulating Multicolor Super-Resolution Imaging Using an RGB Camera

**DOI:** 10.34133/csbj.0162

**Published:** 2026-07-07

**Authors:** Ava E. D. Kelly, John S. H. Danial

**Affiliations:** ^1^ SUPA School of Physics and Astronomy, University of St Andrews, St Andrews, UK.; ^2^ Centre of Biophotonics (CoB), University of St Andrews, St Andrews, UK.

## Abstract

High-order multiplexing in super-resolution microscopy is limited by trade-offs between spectral discrimination, imaging speed, and experimental complexity. Here, we show that red-green-blue (RGB) complementary metal oxide semiconductor (CMOS) cameras provide a simple and scalable solution for multicolor DNA Point Accumulation in Nanoscale Tomography by exploiting their intrinsic spectral sensitivity for statistical fluorophore discrimination. Using a realistic simulation framework incorporating experimentally derived photon budgets, optical response functions, and camera noise, we achieve simultaneous classification of up to 6 fluorophores with a mean precision of ~99%, including perfect discrimination of spectrally overlapping dye pairs, while maintaining an average localization precision of ~3.6 nm and an average localization accuracy of ~32 nm. Performance remains robust to variations in classification thresholds but degrades with reduced photon budgets, and more modestly with increasing fluorophore number, due to spectral overlap and photon noise. The results presented here provide preliminary evidence that RGB detection could support multiplexed super-resolution imaging using a relatively simple and cost-effective experimental configuration. Further studies will be needed to assess its broader applicability and performance relative to conventional spectral imaging approaches.

## Introduction

Single-molecule localization microscopy (SMLM) has revolutionized biological imaging by enabling the organization of multiprotein complexes to be resolved at molecular resolutions, directly in situ [1,2]. This capability has made it possible to visualize flexible assemblies such as clathrin-coated pits [3], filamentous proteins [4], and apoptotic complexes [5] that would typically evade structural characterization using alternative techniques, allowing them to be imaged in their native environments. Despite substantial advances in improving SMLM resolution, achieving rapid, accessible, and high-order multitarget imaging remains a significant challenge.

On the one hand, spectral imaging approaches such as ratiometric imaging using beam-splitting devices are relatively easy to integrate into a wide range of custom and commercial instruments. However, they are typically limited to multiplexing up to 3 targets and often require reduced fields of view (FoVs) [6]. Diffraction gratings [7] and prisms [8,9] can help overcome this limitation, but at the cost of significantly increased experimental complexity, restricting their use to specialist laboratories. While it is in principle possible to combine ratiometric and multidimensional imaging using multiple dichroic beam splitters and cameras that scale with the number of targets, such configurations, though recently demonstrated [10], are both economically and technically challenging to implement.

On the other hand, sequential imaging strategies based on orthogonal chemistries such as DNA Point Accumulation for Imaging in Nanoscale Topography (DNA-PAINT) [11] and its variants, including secondary label-based unlimited multiplexed DNA-PAINT (SUM-PAINT) [12] and fluorogenic labeling with transient adapter-mediated switching for high-throughput DNA-PAINT (FLASH-PAINT) [13], as well as DNA or protein barcoding [14,15] and multiplexing using erasable signals [16], can enable imaging of up to 30 targets within a single FoV. However, these approaches require intricate DNA sequence design where applicable and complex and labor-intensive experimental protocols, and, importantly, suffer from markedly reduced imaging speeds.

More recently, intrinsic photophysical properties of fluorophores such as intensity [17] and fluorescence lifetime [18] have been exploited to distinguish between different labels. Intensity-based discrimination represents the simplest route to rapid, multicolor SMLM, but has so far only been demonstrated using 3 red-emitting dyes. Lifetime-based approaches, while capable of distinguishing up to 8 fluorophores, have been limited to confocal implementations and confocal-type super-resolution modalities rather than wide-field imaging.

Inspired by the human visual system, specifically the role of cone cells in enabling accurate color discrimination, we simulate the performance of industrial red-green-blue (RGB) complementary metal oxide semiconductor (CMOS) cameras for high-throughput, full-color DNA-PAINT imaging. Unlike conventional monochrome sensors, RGB cameras retain sufficient spectral information to enable statistical discrimination between fluorophores, even when their emission spectra overlap, effectively introducing color as an additional measurement dimension. Our simulations demonstrate up to 99% accurate classification of 6 fluorophores imaged simultaneously, paving the way for the experimental realization of straightforward, high-order, multicolor DNA-PAINT, and possibly SMLM, at full imaging throughput.

## Methods

Nine commercially available fluorophores were included in the simulations, with emission maxima ranging from 519 nm (AF488) to 682 nm (CF660R) and experimentally measured photon outputs per 100 ms ranging from 2,073 to 17,653 photons (Supplementary Materials). Photon outputs accounted for differences in fluorophore absorption, given that the excitation source was fixed at 488, 560, or 640 nm, and were derived from experimental measurements of total fluorescence prior to photobleaching and performed using the same buffer (i.e., Trolox) [19]. These fluorophores were chosen to span the spectral range accessible with the 3 excitation wavelengths and to provide a representative set of fluorophores commonly used in multicolor super-resolution experiments.

A virtual microscope was simulated in Python to replicate a typical experimental setup. The optical configuration consisted of a 3-color excitation source, which was directed through a 100×, 1.25 numerical aperture objective lens. Emitted fluorescence was collected by the same objective, passed through a penta-band dichroic mirror (DI01-R405/488/561/635/800, Semrock) and a quad-band emission filter (FF01-446/523/600/677, Semrock), and finally focused via a 70-mm tube lens onto an RGB camera (Blackfly S BFS-U3-32S4C-BD, FLIR) to yield a final pixel size of 98.6 nm. The spectral response of each component, including the excitation sources, dichroic, emission filter, and camera quantum efficiency, was measured experimentally and combined with each fluorophore’s emission spectrum to generate a single compounded spectral response curve for each fluorophore. This curve determined the fraction of photons detected in each of the red, green, and blue channels of the RGB camera for a given fluorophore.

Photon outputs for each emitter were sampled from a Poisson distribution around the experimentally determined values [19] to model realistic photophysical variation [20]. The point spread function was modeled as a Gaussian of standard deviation σ, defined by the objective numerical aperture (*NA*) and the fluorophore emission wavelength (λ), according to:

$$\sigma =\frac{\lambda }{4\mathrm{NA}}$$ (1)

The resulting photon distributions were converted to electron counts and digitized into analog-to-digital units (ADUs) using experimentally measured camera parameter maps [21], accounting for pixel-wise variations in CMOS sensors: a baseline offset map (median: 128.2 ADU), a dark current map (median: 0.6 e^−^/pixel/s), a noise map accounting for thermal and read noise (median: 13.6 ADU), and a gain map (median: 2.0 ADU/e^−^), with shot noise added to replicate realistic measurement conditions. Signals were then distributed across the RGB channels according to the compounded spectral response for each fluorophore, creating simulated images that closely matched those obtained in physical experiments.

Simulated images were generated with a 512 × 512-pixel FoV (50.5 × 50.5 μm), containing 250 randomly distributed 2 × 3 DNA origami with binding sites separated by 20-nm distances. Per dye, 100 binding events per frame were simulated over 1,000 frames. For each dye, 2 sets of 1,000-frame stacks were simulated: one used to build RGB intensity histograms for dye fingerprinting as described below, and one used to assess classification performance.

We firstly processed the simulated images using the ImageJ plugin ThunderSTORM [22]. A B-Spline wavelet filter was applied with order 3 and scale 2. Approximate localization of molecules was performed via local maximum detection, using an intensity threshold equal to the standard deviation of the filtered image. Subpixel localization was performed by maximum likelihood fitting of a Gaussian, with a fitting radius of 3 pixels and an initial sigma of 1 pixel. Resulting localizations were filtered to retain PSF widths between 90 and 140 nm, and further filtered by intensity and localization precision, with thresholds adjusted per dye to remove outliers and signals arising from overlapping localizations (Supplementary Materials).

After localization filtering, mean red, green, and blue pixel intensities were extracted from a 5 × 5-pixel region centered on the retained localizations, and histograms of these intensities were compiled to characterize the expected intensity distributions in each color channel. Identification regions were defined for each fluorophore to encompass 60% of the intensity values, although this fraction could be adjusted to optimize either specificity or sensitivity. An emitter was assigned to a fluorophore if its mean R and G intensities fell exclusively within the identification regions of that fluorophore; emitters falling outside all identification regions or within the identification regions for more than one fluorophore were labeled as unknown.

To assess the performance of this classification method, we defined 2 metrics. Given *C* correct identifications, *I* incorrect identifications, and *U* detections classified as unknown, classification precision (*P*) is defined as:

$$P=\frac{C}{C+I}$$ (2)

and abstention rate (*A*) as:

$$A=\frac{U}{U+I+C}$$ (3)

We also assessed localization precision and accuracy for each dye. Localization precision was calculated using measured photon outputs and PSF widths obtained in ThunderSTORM, following localization filtering (Supplementary Materials). Localization accuracy was assessed by simulating DNA origami images with larger binding site separations of 100 nm. After applying the same localization and filtering procedure used for the 20-nm origami (Supplementary Materials), super-resolved images were relocalized in ThunderSTORM using maximum likelihood Gaussian fitting to extract the spatial spread of repeated localizations around each binding site, taken as a measure of localization accuracy.

## Results

**Fig. 1.**
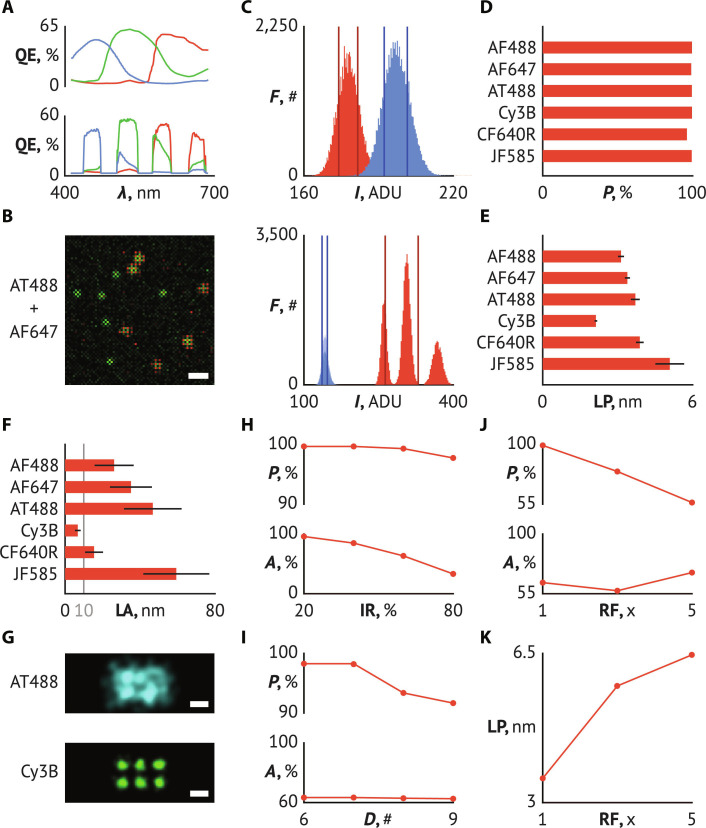
(A) The averaged spectral response of the blue, green, and red pixels of the camera (top) is compounded with the spectral response of the dichroic and emission filters (bottom). (B) Single emitters are convoluted with a Gaussian Point Spread Function (PSF) whose width is calculated using each fluorophore’s emission wavelength and the objective’s numerical aperture. Example image showing single ATTO 488 and Alexa 647 dyes simulated on a camera with an RGB Bayer filter. Scale bar = 1 μm. (C) The RGB intensity of each emitter is measured and plotted as histograms. An identification region (IR) is defined to include 60% of the intensities. An unknown emitter is assigned to a fluorophore if its RGB values fall within that fluorophore’s IR; otherwise, it is rejected. Intensities (*I*) of ATTO 488 (blue) and Alexa 647 (red) and their respective IRs are plotted in the green (top) and red (bottom) channels. (D) Classification precision (*P*), calculated as the number of correct identifications (*C*) divided by the sum of *C* and the number of incorrect identifications (*I*), is quantified for 6 dyes with realistic photon budgets extracted from Ref. [19] for DNA-PAINT experiments. Our results indicate an average classification precision of 99.3%. (E) Localization precision (LP), calculated from the measured photon count and PSF width of each localization in ThunderSTORM, is not worsened using an RGB camera, where Cy3B is localizable with a precision of 2.14 nm. (F) Localization accuracy (LA) was measured by simulating DNA origami with 100-nm separation of binding sites and measuring the spread of repeated localizations around each binding site. Our results indicate an average localization accuracy of 31.76 nm. (G) Super-resolved images of simulated DNA-origami structures for 2 example dyes, ATTO 488 (top) and Cy3B (bottom). Binding sites separated by 20 nm are clearly resolved with Cy3B but not with ATTO 488, in agreement with our measured localization accuracies. Scale bar = 20 nm. (H) *P* and the abstention rate (*A*), calculated as unknown identifications (*U*) divided by *C* + *I* + *U*, quantified against width of IR. (I) *P* and *A* quantified against the number of possible dyes (D). (J) *P* and *A* quantified against the average photon budget reduction factor (RF), where 1 represents an average of 7,035 photons and 5 represents an average of 1,407 photons across the 6 dyes. (K) LP quantified against RF.

Using experimentally determined photon budgets reported previously [19] (mean photon count of 7,035 for 100-ms exposures, Supplementary Materials) and defining an identification region spanning 60% of the spectral space, we first evaluated the performance of our classification framework across 6 dyes: Alexa Fluor 488 (AF488), Alexa Fluor 647 (AF647), ATTO 488 (AT488), Cy3B, CF640R, and JF585. Under these conditions, the system achieved a high mean classification precision of approximately 99%. Notably, 5 dyes (AF488, AT488, AF647, Cy3B, and JF585) were classified with 100% precision, indicating near-perfect separability within the defined identification space (Fig. 1 and Supplementary Materials).

In parallel, we quantified the localization precision and accuracy achievable using an RGB camera under the same experimental constraints. Across those 6 dyes, an average localization precision of 3.57 nm was obtained, with an average localization accuracy of 31.76 nm. Among these, Cy3B, which is commonly employed in DNA-PAINT experiments, exhibited particularly high performance, achieving a localization precision of 2.14 nm and a localization accuracy of 6.80 nm, consistent with its favorable photophysical properties (Fig. 1 and Supplementary Materials).

Having established baseline performance under realistic conditions, we next systematically challenged the framework by varying 2 key parameters: (a) the width of the identification region (ranging from 20% to 80%), and (b) the number of distinguishable dyes (increasing from 6 to 9). Varying the identification region width had a relatively minor impact on classification precision, which decreased only modestly from 99.67% to 97.74%. However, this came at the expense of a substantial increase in the abstention rate (defined as the fraction of “unknown” classifications), which increased from 33.16% to 95.80% as the identification region was narrowed from 80% to 20% (Fig. 1 and Supplementary Materials). This behavior reflects a more conservative classification regime, in which the system increasingly rejects ambiguous detections rather than risking misclassification.

In contrast, both classification precision and abstention rate remained relatively robust to changes in dye number. As the number of dyes was varied from 6 to 9, the abstention rate remained stable at approximately 60% (ranged from 62.57% to 63.36%), while the classification precision decreased moderately from 99.31% to 96.67%, indicating increased overlap in the spectral signatures and a higher likelihood of incorrect assignments when more dyes are included (Fig. 1 and Supplementary Materials).

Finally, we investigated the impact of reduced photon budgets to assess the feasibility of using RGB-based classification and localization in photon-limited applications. A 5-fold reduction in the mean photon budget resulted in a moderate increase in the abstention rate (from 63.36% to 70.93%), but a more pronounced degradation in classification precision, which dropped from 99.31% to 56.24% (Fig. 1 and Supplementary Materials). In parallel, localization precision deteriorated from 3.57 to 6.47 nm (Fig. 1 and Supplementary Materials).

## Conclusion

Although RGB and RGBW cameras have previously been demonstrated for two-color SMLM [23,24], we show here for the first time, through computational analysis, that RGB cameras enable high-classification precision for 6 to 9 dyes imaged simultaneously. Notably, even spectrally proximate dye pairs such as AT488 and AF488, or Cy3B and JF585, can be discriminated with 100% precision, highlighting the resolving capability of this approach despite the limited spectral information available from RGB detection.

Under low photon budget conditions, classification precision decreases, as expected. This reduction can be partially mitigated by limiting the number of dyes included in the analysis. At low photon counts, particularly for dim fluorophores, a substantial fraction of simulated emitters is rejected during preprocessing (Supplementary Materials). This effect arises from an apparent broadening of the point spread function, which occurs when emitter intensities approach the camera noise floor and reduces both detectability and fitting reliability. These observations highlight the sensitivity of both classification and localization performance to photon statistics, while also demonstrating that meaningful performance can still be achieved under photon-limited conditions.

Although the simulations capture many aspects of a realistic experimental system, certain optical aberrations, including spherical and chromatic aberrations, were not explicitly modeled. This simplification is justified by the high correction quality of modern objective and tube lens systems, which minimize such aberrations under typical imaging conditions. In addition, while the simulations were performed using a commonly employed combination of dichroic and emission filters for multicolor single-molecule experiments, alternative filter sets may further improve both fluorophore classification and localization precision. Our open-source code allows users to readily incorporate alternative dichroic and emission filter spectral data, facilitating the evaluation and optimization of different optical configurations for specific fluorophore combinations and experimental requirements.

Furthermore, we acknowledge that our simulations were limited to surface-bound or surface-proximal structures imaged under total internal reflection illumination using carefully tuned laser powers, with DNA-PAINT used to spatially separate nearby single molecules. Consequently, the extent to which these findings can be generalized to other SMLM modalities, particularly intracellular imaging applications, remains unclear and will require experimental verification.

We also note that, even within this restricted simulation framework, certain photophysical artifacts could not be readily modeled. These include effects arising from simultaneous high-power tri-laser illumination, such as accelerated photobleaching through higher-order triplet-state processes and increased photo-induced destruction of docking sites. As these phenomena may influence imaging performance and fluorophore classification accuracy, their impact warrants further experimental investigation and validation.

Additionally, we adopted a simple fluorophore classification scheme in which the red and green intensities of each detected emitter were required to fall within the identification region of a single dye; emitters that could not be assigned with confidence were discarded. This approach was chosen primarily to demonstrate the feasibility of fluorophore classification using RGB imaging. However, it inevitably results in a proportion of fluorophores remaining unclassified and therefore being excluded from the final dataset. Under highly narrow identification regions, the proportion of unclassified fluorophores can exceed 90%, necessitating longer acquisition times and diminishing the advantages of simultaneous multicolor detection. However, we have demonstrated that such conservative identification windows offer little benefit to classification precision; precisions of 97% to 99% can be achieved using wider identification regions while reducing the proportion of unclassified fluorophores to approximately 30% to 60%.

Alternative classification strategies could be explored to reduce the number of discarded emitters, including regression-based methods, machine learning approaches, or classification based on the red-to-green intensity ratio rather than the individual intensity values alone. While it is plausible that more sophisticated and optimized algorithms could improve both localization precision and data retention, this remains speculative and will require dedicated investigation and experimental validation. To facilitate such developments, we provide open-source, well-documented code that will enable the community to explore alternative classification approaches and develop optimized algorithms without compromising sensitivity or specificity.

We also note that the localization precisions reported here are calculated according to an estimate of the Cramér–Rao lower bound. This may partly account for the discrepancy between these and the localization accuracies observed, which ranged from ~6.8 to ~59.6 nm across dyes. This meant that 20-nm origami structures could be resolved for only a subset of dyes tested. Notably, the observed localization accuracies do not follow a monotonic relationship with dye brightness. This may be due to the unequal ratio of green to red pixels on the RGB camera sensor resulting in undersampling of red-channel emitters, reducing localization accuracy for red dyes compared to green dyes of the same brightness. Within the specific context of DNA-PAINT, these localization accuracies can be readily improved using Gaussian Mixture Modeling [25].

Finally, our computational analysis suggests that high-order multicolor DNA-PAINT may be achievable using an industrial RGB CMOS camera. While these findings indicate the potential for a cost-effective alternative to conventional detection systems, extensive experimental validation will be required to assess performance under practical imaging conditions. Furthermore, scientific-grade RGB CMOS cameras, which typically offer higher quantum efficiency and lower noise characteristics, could provide additional improvements in localization precision; however, these predictions likewise require rigorous experimental verification.

## Data Availability

All datasets are publicly available at https://doi.org/10.5281/zenodo.20764371. All codes are publicly available on https://github.com/aedk02/RGB_Simulator.
